# Body Position Influences Which Neural Structures Are Recruited by Lumbar Transcutaneous Spinal Cord Stimulation

**DOI:** 10.1371/journal.pone.0147479

**Published:** 2016-01-21

**Authors:** Simon M. Danner, Matthias Krenn, Ursula S. Hofstoetter, Andrea Toth, Winfried Mayr, Karen Minassian

**Affiliations:** 1 Department of Neurobiology and Anatomy, Drexel University College of Medicine, Philadelphia, PA, United States of America; 2 Institute for Analysis and Scientific Computing, Vienna University of Technology, Vienna, Austria; 3 Center for Medical Physics and Biomedical Engineering, Medical University of Vienna, Vienna, Austria; Heidelberg University Hospital, GERMANY

## Abstract

Transcutaneous stimulation of the human lumbosacral spinal cord is used to evoke spinal reflexes and to neuromodulate altered sensorimotor function following spinal cord injury. Both applications require the reliable stimulation of afferent posterior root fibers. Yet under certain circumstances, efferent anterior root fibers can be co-activated. We hypothesized that body position influences the preferential stimulation of sensory or motor fibers. Stimulus-triggered responses to transcutaneous spinal cord stimulation were recorded using surface-electromyography from quadriceps, hamstrings, tibialis anterior, and triceps surae muscles in 10 individuals with intact nervous systems in the supine, standing and prone positions. Single and paired (30-ms inter-stimulus intervals) biphasic stimulation pulses were applied through surface electrodes placed on the skin between the T11 and T12 inter-spinous processes referenced to electrodes on the abdomen. The paired stimulation was applied to evaluate the origin of the evoked electromyographic response; trans-synaptic responses would be suppressed whereas direct efferent responses would almost retain their amplitude. We found that responses to the second stimulus were decreased to 14%±5% of the amplitude of the response to the initial pulse in the supine position across muscles, to 30%±5% in the standing, and to only 80%±5% in the prone position. Response thresholds were lowest during standing and highest in the prone position and response amplitudes were largest in the supine and smallest in the prone position. The responses obtained in the supine and standing positions likely resulted from selective stimulation of sensory fibers while concomitant motor-fiber stimulation occurred in the prone position. We assume that changes of root-fiber paths within the generated electric field when in the prone position increase the stimulation thresholds of posterior above those of anterior root fibers. Thus, we recommend conducting spinal reflex or neuromodulation studies with subjects lying supine or in an upright position, as in standing or stepping.

## Introduction

Electrical stimulation with skin-attached electrodes placed paravertebrally at the level of the lumbosacral spinal cord aims at selectively depolarizing sensory fibers in the posterior roots [1]. The resulting reflexes, thought to share some physiological similarity with the H reflex [2], can be evoked by a single pulse in virtually all lower limb muscle groups and have been named posterior root-muscle (PRM) reflexes [1,3]. The reliable and simultaneous stimulation of afferents related to multiple dermatomes and myotomes provides the opportunity for use in neuromodulation applications [4].

Recently, and in parallel with its epidural counterpart [5–7], transcutaneous spinal cord stimulation has experienced a surge of interest: PRM reflex modulation was studied in able-bodied individuals during gait [8] and specified motor tasks [9] as well as in individuals with motor complete spinal cord injury during assisted treadmill stepping [10]; the relationship between vertebral electrode position and root stimulation was investigated [11–14]; the interaction of PRM reflexes with motor evoked potentials was characterized [15–17]; its physiological interaction with the soleus H reflex was investigated [2,18]; and, similar to epidural lumbar spinal cord stimulation [7,19,20], exploratory studies using tonic transcutaneous stimulation showed its potential for the control of spinal spasticity [21], modification of altered gait [22,23], and activation of lumbar locomotor pattern generating networks after spinal cord injury [24].

Transcutaneous elicitation of PRM reflexes was initially demonstrated in the supine position [1]. Stimulation was applied in an upright position for studying the modification of motoneuron excitability during treadmill stepping [8,10] and for neuromodulation of gait after complete [24] and incomplete spinal cord injury [22]. Also, subjects were studied in prone [25–27], semi-prone seated [18], and seated [2,13] positions, presumably adapted from conventional body positioning for H reflex studies and paravertebral magnetic stimulation. In supine, standing and stepping the responses were suppressed by tendon vibration and repetitive stimulation or were modulated by volitional motor tasks [1,8,9], showing the hallmark properties of monosynaptic reflexes [28]. During semi-prone sitting they were not modified by repetitive stimulation and presumed to be mostly caused by activation of motor fibers [18]. Further, direct responses to anterior root motor fiber stimulation can be readily obtained when stimulation is applied in the prone position [25,26].

We hypothesized that the body position determines the relative thresholds of sensory compared to motor fibers and thus the nature of the evoked responses. We applied transcutaneous spinal cord stimulation with single and paired pulses in the supine, standing and prone positions and recorded the evoked electromyographic (EMG) responses from the thigh and leg muscle groups. Paired-pulse paradigms are a tool to distinguish between reflex and direct motor fiber responses, as second reflex responses appear suppressed by post-activation depression, whereas motor fibers react with two equal responses. We show that the assumed body position strongly affects the responses: in the supine and standing positions they are of reflex nature while in the prone position they are caused by concomitant motor fiber stimulation.

## Materials and Methods

### Subjects

This study was approved by the Ethics Committee of the City of Vienna and was conducted in compliance with the Declaration of Helsinki [29]. Prior to participation each subject signed an informed consent form. Ten able-bodied adults (6 female) aged 18–34 years (26.5 ± 4.8 years) with intact nervous systems and without orthopedic disorders participated in this study.

### Stimulation setup

Three interconnected silver-silver chloride electrodes (1 cm diameter, T-60, Leonhard Lang GmbH, Innsbruck, Austria) were placed horizontally on the paravertebral skin between the T11 and T12 spinous processes. The central electrode was medially placed and the other two left and right to it with a center-to-center distance of 2 cm. Two interconnected rectangular self-adhesive electrodes (8x13 cm, STIMEX, schwa-medico, Ehringshausen, Germany) were placed on the abdomen, forming a medially positioned transversely oriented 16x13 cm reference electrode. Symmetric biphasic stimulation pulses with a width of 1 ms per phase were delivered by a current-controlled stimulator (Stimulette r2x+, Dr. Schuhfried Medizintechnik GmbH, Vienna, Austria). The paravertebral electrodes acted in the first phase as cathodes and in the second as anodes.

### Data acquisition

EMG recordings from quadriceps, hamstrings, tibialis anterior, and triceps surae muscles were acquired bilaterally using pairs of silver-silver chloride recording electrodes (Intec Medizintechnik GmbH, Klagenfurt, Austria), each placed centrally on the muscle bellies and oriented along the long axis of the muscles with an inter-electrode distance of 3 cm [30]. EMG signals were amplified with a gain of 600, filtered to a bandwidth of 10–600 Hz, and digitized at 10,000 samples per second and channel with a USB-NI 6261 data acquisition card (National Instruments Inc., Austin, TX, USA) and recorded using DasyLab 11.0 (Measurement Computing Corporation, Norton, MA, USA).

### Stimulation protocol

Stimulation was applied with subjects in supine, standing and prone positions. In the supine and prone positions, subjects lay flat on their back or front, respectively, with a neutral head position and extended legs. In the standing condition, they assumed a straight upright position referenced to a wall, without contact or external support. In all positions the arms were held parallel to the torso. If movement or EMG activity was observed, stimulation was postponed for 10 s. In the standing position, tonic EMG activity was minimized with feedback from the examiners and stimulation was postponed when phasic EMG activity related to postural corrections occurred. For each body position, a paired-pulse with 30 ms inter-stimulus interval and three single pulses were applied. Stimulation intensity was increased in 5-mA increments starting at 5 mA up to 125 mA or the individual maximum comfortable intensity. The interval between consecutive single or paired pulses was at least 7 s.

### Data analysis

Data analysis was performed using MATLAB R2012b (Mathworks, Natwick, MA) and statistical analysis using IBM SPSS Statistics 22.0 (IBM Corporation, Armonk, New York, NY) for Mac OS X 10.9.3. For the first responses the mean peak-to-peak amplitude of the three responses to the single stimuli and for the second the amplitude of the second response to the paired stimulus were used for further analysis. In one subject the paired-pulses were applied with an inter-stimulus interval of 50 ms and the respective second responses were hence excluded from the analysis (declared as missing data). The threshold intensity for each individual muscle was defined as the lowest intensity level at which the peak-to-peak amplitude of the first response surpassed 50 μV. In case this threshold intensity was not reached in a muscle group with the maximum applicable intensity it was defined as being 5 mA larger than the highest tested intensity. The ratios of the amplitudes of the second to the first responses were calculated, or declared as missing data if first responses were absent.

Response amplitudes were compared at the highest comfortable stimulation intensity applied across all body positions in a given subject or at a maximum of 100 mA (100 mA in six, 70 mA in two, and 95 and 85 mA in one subject each). Two two-factorial repeated measurement analyses of variance (ANOVA) with full factorial designs were calculated to investigate the effect of body position and muscle group on the amplitude of the first response and the threshold intensity. Recordings from each leg were treated as a single case. The assumption of sphericity was tested with Mauchly’s test, and if voided Greenhouse-Geisser correction was applied. To adjust for missing data in the ratios of second to first responses (i.e., when threshold was not reached), a generalized linear mixed model with Satterthwaite correction was calculated for the effect of body position and muscle group (repeated measures) on this ratio. Leg and subject were modeled as hierarchical random effects. Covariance type was determined by minimizing the Akaike Information Criterion. For both, the repeated measures ANOVA and the mixed model, the effect sizes were reported by the partial eta-squared ($\eta_{p}^{2}$). Categorical data were analyzed using Pearson's χ^2^ test; the assumption that the expected count for every cell is larger than 5 was checked. All post-hoc tests were Bonferroni corrected and an α-error of P < .05 was regarded as significant.

## Results

**Fig 1:**
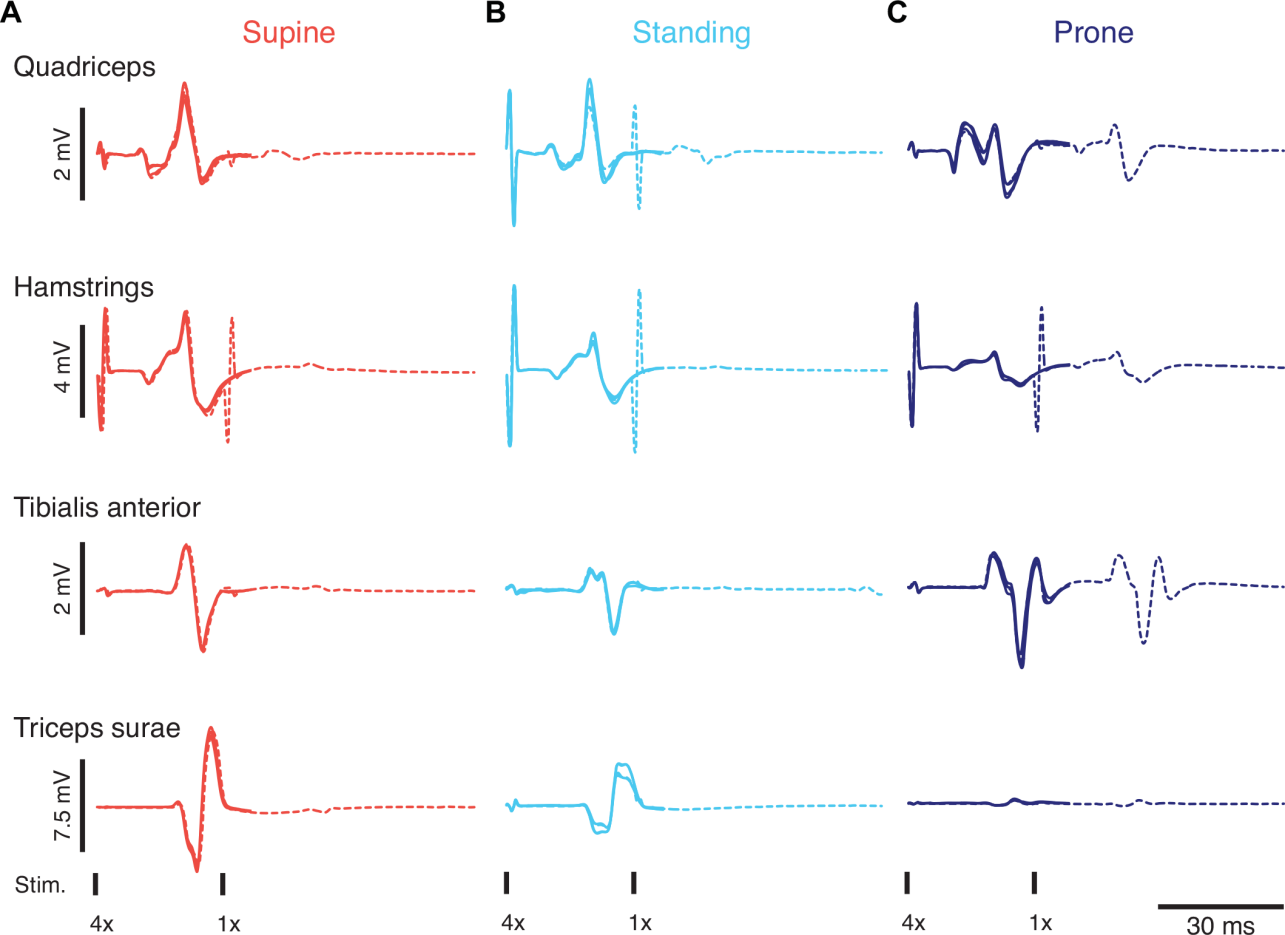
Exemplary results. Stimulus time-locked EMG responses elicited in the supine (A), standing (B), and prone position (C) with unchanged stimulation parameters. The responses were largest in supine and smallest in prone position with the exception of tibialis anterior. Three responses to single stimuli (solid lines) and responses to a pair of pulses with 30 ms inter-stimulus interval (dashed lines) are shown superimposed for each body position and muscle studied. The second responses were suppressed in the supine position and during standing, while in the prone position they almost retained the size of the first responses. All responses were elicited at 100 mA and were derived from the same subject.

**Fig 2:**
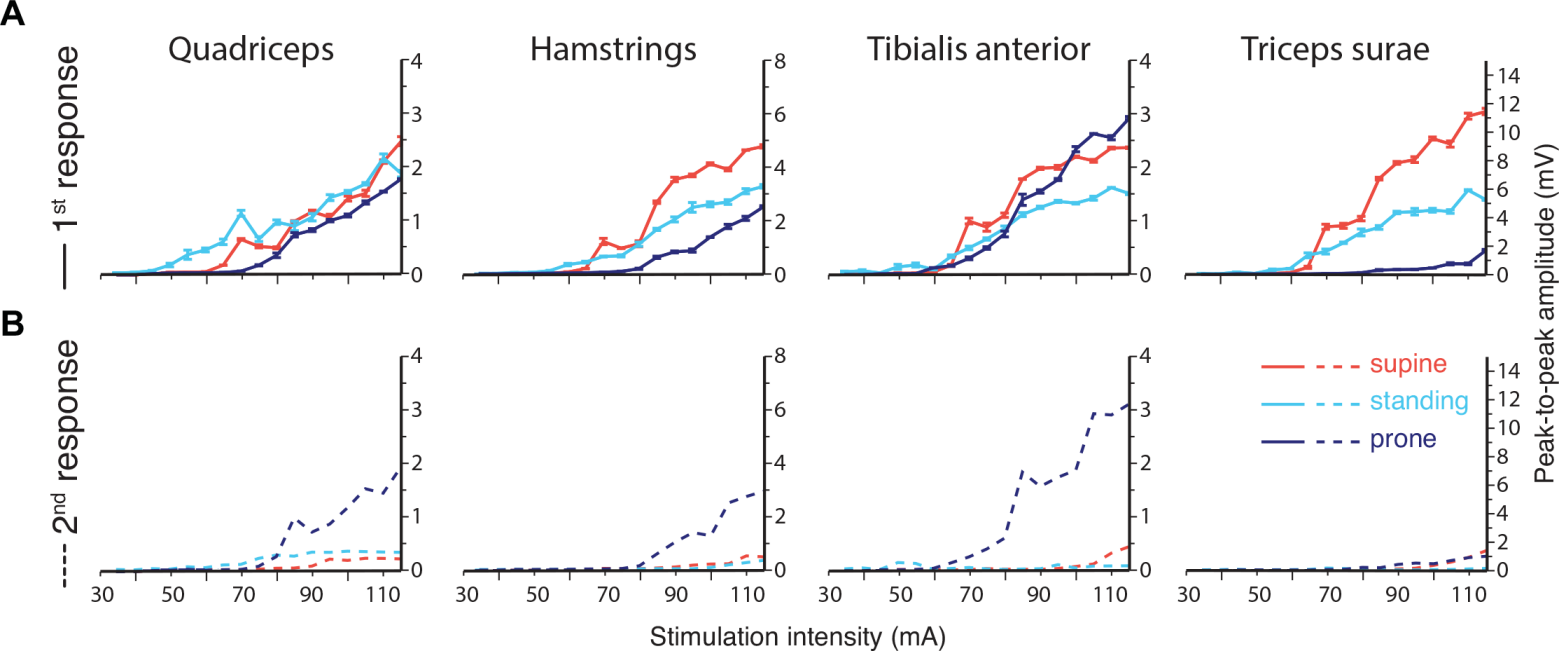
Recruitment curves. The peak-to-peak amplitudes of the first (A) and second responses (B) of the same subject as in Fig 1 are illustrated in the three body positions (red: supine, cyan: standing, dark blue: prone) as a function of the applied stimulation intensity for all muscle groups. Second responses were largest in prone position. The threshold intensity was lowest while standing.

Representative EMG responses from a single subject evoked by transcutaneous spinal cord stimulation at 100 mA in the three body positions are shown in Fig 1. The corresponding recruitment curves (i.e., functions of response amplitude vs. stimulation intensity) are depicted in Fig 2. First responses were generally largest in the supine and smallest in the prone position while the opposite was the case for the second responses (Fig 1). During standing, responses were evoked at lower intensities than in the other body positions, but attained lower amplitudes than in the supine position (Fig 2). In the prone position the amplitudes of the second response matched those of the first across all intensities, while in the other positions they were markedly reduced. In the following we present the statistical results across all subjects.

### Response amplitudes

**Fig 3:**
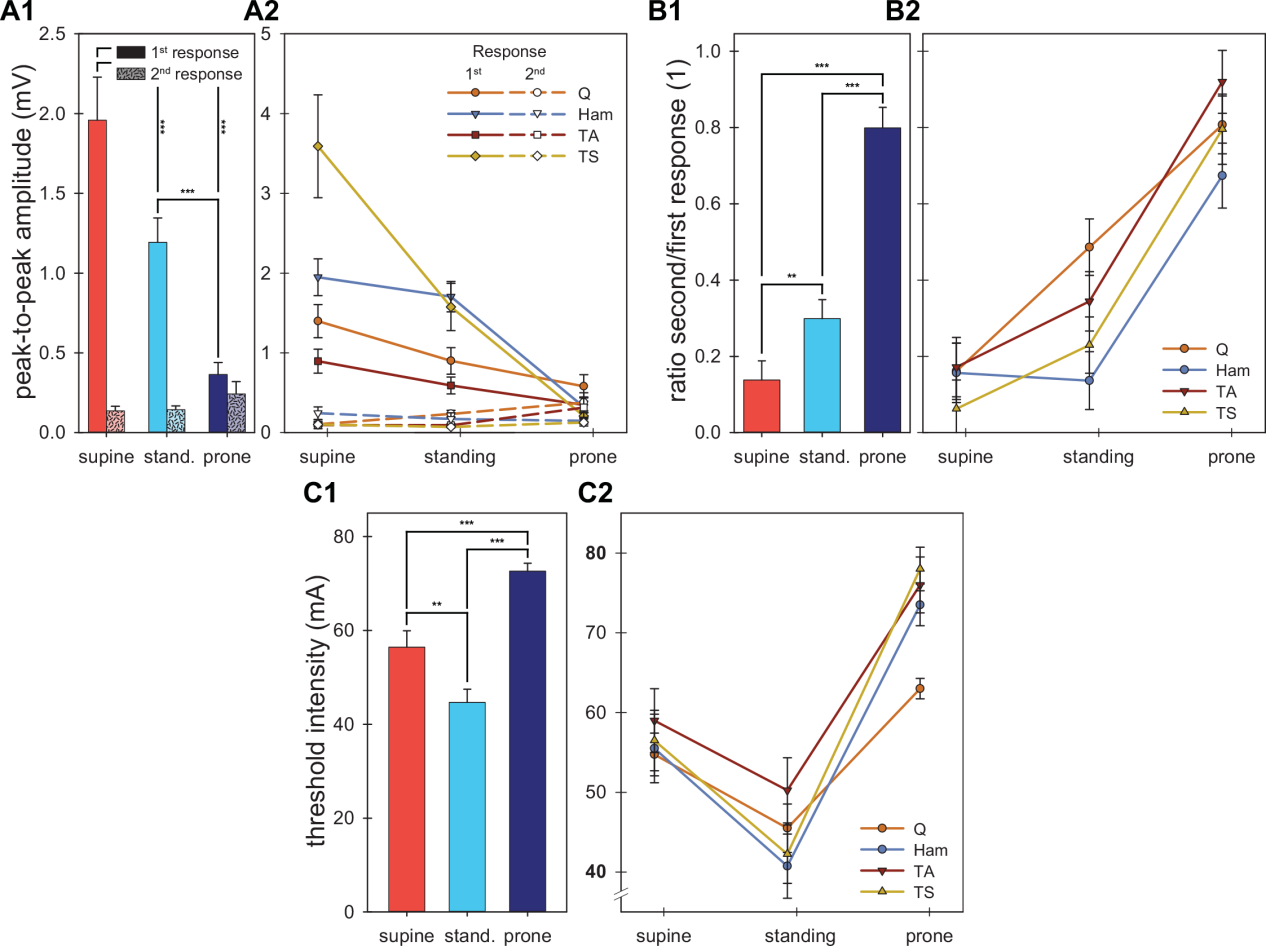
Group results. Means and marginal means are depicted for the peak-to-peak amplitudes of the first and second responses (A), the ratio between the second and first responses (B), and the threshold intensity (C). (A1–C1) Marginal means for the fixed effect body position. (A2–C2) Means for the interaction effect between body position and muscle group. Amplitudes of the first responses were largest in the supine, followed by standing and prone position (A1). The opposite was the case for the second responses (A1). Threshold intensities were lowest in standing and largest in the prone position (B1). Significant results of the pairwise post-hoc tests between the body positions are indicated with asterisks for the main effects. Error bars indicate standard errors. Q: quadriceps, Ham: hamstrings, stand.: standing, TA: triceps surae, TS: triceps surae, *: P<0.05, **: P<0.01, ***: P<0.001.

The repeated measures ANOVA of the mean peak-to-peak amplitude of the first responses between body position and muscle groups showed several significant results (Fig 3A). Mauchly’s test indicated that the assumption of sphericity was violated for the main effects of body position [χ^2^(2) = 8.314, P = .016] and muscle group, [χ^2^(5) = 27.128, P < .001] as well as for their interaction effect [χ^2^(20) = 102.007, P < .001]. Thus, the degrees of freedom were corrected using Greenhouse-Geisser estimates of sphericity (ε = .730 for the main effect of body position, ε = .553 for the main effect of muscle group and ε = .265 for their interaction effect). There was a significant main effect of the body position on the amplitude of the first response [supine: 1.96±0.27 mV (mean ± standard error), standing: 1.20±0.15 mV, prone: 0.37±0.08 mV; F_1.460,27.739_ = 43.060, P < .001, $\eta_{p}^{2}$ = .694; Fig 3A1]. Across muscles, the first responses were significantly largest in supine and smallest in prone position (all pairwise post-hoc tests P < .001). There was also a significant main effect of the muscle group on the amplitude of the first response (quadriceps: 0.96±0.15 mV, hamstrings: 1.33±0.14 mV, tibialis anterior: 0.61±0.10 mV; triceps surae: 1.79±0.32 mV F_1.659,31.520_ = 14.221, P < .001, $\eta_{p}^{2}$ = .428). Across body positions, paired post-hoc tests showed that the first responses in triceps surae and hamstrings were significantly larger than in quadriceps and tibialis anterior. Triceps surae had the largest and tibialis anterior the smallest responses. Finally, the interaction effect between muscle group and body position was highly significant (F_1.592,30.256_ = 16.078, P < .001, $\eta_{p}^{2}$ = .458; Fig 3A2), and showed that the relationship between the response amplitudes differed between body positions. In supine position responses of different amplitudes were evoked in the various muscles, while in prone the responses of all muscles had similar amplitudes. Between supine and standing the amplitude decrease of triceps surae responses was strongest in comparison to that of the other muscles. In each muscle group, responses were significantly larger in supine than in prone position.

The mixed generalized linear model analysis of the ratio of the amplitudes of the second to the first responses (Fig 3B) was highly significant (F_11,128_ = 16.753, P < .001, $\eta_{p}^{2}$ = .591) with the strongest effect between the body positions (supine: 0.14±0.05, standing: 0.30±0.05, prone: 0.80±0.06; F_2,63_ = 73.729, P < .001, $\eta_{p}^{2}$ = .700; Fig 3B1). Across muscles, the ratios were largest in prone, followed by standing, and supine position. All pairwise post-hoc tests were significant (all P<0.01). The ratios also differed between the muscle groups across body positions (quadriceps: 0.49±0.05; hamstrings: 0.32±0.05, tibialis anterior: 0.48±0.05, triceps surae: 0.36±0.05; F_3,129_ = 4.919, P = .003, $\eta_{p}^{2}$ = .102), with lower values for hamstrings than for quadriceps and tibialis anterior. There was no significant interaction effect of the muscle groups and body positions (F_6,128_ = 1.729, P = .119, $\eta_{p}^{2}$ = .075; Fig 3B2). Hamstrings was the only muscle group without increase of the ratio from supine to standing.

Across subjects, a total of 2 (3.0%) muscles in supine position, 16 (23.2%) during standing and 42 (76.4%) in the prone position responded with second responses larger than half the size of the respective first responses [χ^2^(2) = 78.877, P < .001]. Thus, the odds that in prone the second response was suppressed by less than 50% compared to the first response was 25.5 and 3.3 times higher than those in supine and standing, respectively. This general relation was present in all muscle groups (all P < .001). There were, however, no significant differences between the muscle groups [across the body positions; quadriceps: 22, 41.5%, hamstrings: 9, 19.6%, tibialis anterior: 16, 34.8%, triceps surae: 13, 28.3%, χ^2^(3) = 5.959, P = .114].

### Threshold intensity

A two-way repeated measures ANOVA of the effect of body position and muscle group on the threshold intensity yielded several significant results (Fig 3C). Mauchly’s test indicated that the assumption of sphericity was neither violated for the main effects [body position: χ^2^(2) = 2.332, P = .312; muscle group: χ^2^(5) = 4.070, P = .540] nor the interaction effect [χ^2^(20) = 27.120, P = .138]. There was a significant main effect of body position (supine: 56.4±3.5 mA, standing 44.7±2.8 mA, prone: 72.6±1.7 mA; F_2,38_ = 45.776, P < .001, $\eta_{p}^{2}$ = .707; Fig 3C1). Across muscles, thresholds were lowest during standing, followed by supine and prone position, all pairwise post-hoc tests were significant (all P < .01). The main effect muscle group was significant (quadriceps: 54.4±1.6 mA, hamstrings: 56.6±2.7 mA, tibialis anterior: 61.8±3.1 mA, triceps surae: 58.9±2.4 mA; F_3,57_ = 4.756, P = .005, $\eta_{p}^{2}$ = .200). Across body positions, quadriceps had lower thresholds than tibialis anterior (P = .034). There was a significant interaction effect (F_6,114_ = 3.740, P = .002, $\eta_{p}^{2}$ = .164; Fig 3C2), suggesting that the thresholds of hamstrings and triceps surae changed more between the body positions than those of quadriceps and tibialis anterior (the two muscles with the lower amplitudes of the first and higher ratios of second to first responses). The threshold intensity was reached in all subjects and muscles in supine and standing positions while it was not reached in 5 cases (muscles) in the prone position. Since these missing data points were defined as the highest applied stimulation intensity plus 5 mA (see methods) the threshold in the prone position was underestimated.

## Discussion

Body position strongly affected the response to transcutaneous root stimulation. Suppression of the second response to a pair of stimuli was strong in supine and standing and almost absent when prone. The first responses were largest in the supine and smallest in the prone position. Thresholds for evoking a response independent of its origin were lowest while standing and highest in the prone position.

### Response nature

Responses with similar latencies can result from sensory (posterior root), motor (anterior root) or mixed sensory-motor fiber stimulation when the stimulation site is close to the spinal cord because of the minimal differences in the respective conduction times and the short synaptic delay. Therefore, paired-pulse paradigms have been used to examine the nature of these responses in various studies [1,2,8] where PRM reflexes were also termed multisegmental monosynaptic responses [8] and root evoked potentials [2] by others. Responses to direct stimulation of the motor fibers in the anterior roots must be physiologically similar to the M-waves evoked in peripheral nerves that largely retain their amplitude when repetitively elicited [31–33]. Trans-synaptic responses are subject to additional, central spinal mechanism reducing the reflex output size when repetitively activated. Pre-synaptic mechanism may involve a decreased probability of neurotransmitter release from previously active Ia-afferent terminals as well as presynaptic inhibition of the Ia terminals following the afferent input evoked by a preceding stimulus [28]. Post-synaptic effects would include recurrent inhibition through the preceding, reflexive activation of motoneurons [28] and further inhibitory effects may arise from the synchronous stimulation of afferents of several posterior roots bilaterally, the resulting homo- and heteronymous inputs and the recruitment of a wide range of interneurons [2,34,35].

The degree of reflex attenuation decreases, at a given inter-stimulus interval, with increasing size of the unconditioned reflex [36–38], because large reflexes are less prone to be modified by conditioning input [28]. Thus, the presence of a second response following a large first response does not necessarily mean that motor fibers were electrically stimulated, but may result from the fast recovery of large-amplitude reflexes. Here, the ratio of the second to the first response in prone position was not only close to 1, but also occurred with considerably smaller first responses (that as a reflex would be more likely suppressed) than in the other body positions. Thus, the responses in the prone position were predominantly caused by direct electrical stimulation of motor fibers, yet concomitant activation of some sensory fibers cannot be excluded [18]. The large first responses together with almost absent second responses in supine and standing positions suggest predominant stimulation of sensory fibers. Thus, we demonstrated that the body position not only influences the amplitude of the responses but also whether they resulted from sensory or motor stimulation.

The interpretation of the responses obtained in the supine and standing positions are in accordance with those of previous studies: responses are suppressed by tendon vibration and modulated by volitional motor tasks in supine position [1], as well as by postural tasks [9] and treadmill stepping [8]; stimulating over the cauda equina in supine position increases the response latency in comparison to the T11–T12 electrode position [1]; and the responses in supine position have similar latencies and shapes to PRM reflexes elicited by epidural stimulation of the posterior lumbar spinal cord [39], which are also subjected to post-activation depression when repetitively evoked [37]. Concomitant motor fiber activation has been described in a semi-prone seated position [18]. The prone position has been used for electrical stimulation of anterior roots to assess motor nerve pathologies [25,26].

The information gained from the response thresholds must be carefully interpreted, because whether afferents or efferents were primarily activated could be different between the body positions. However, the identification of the response nature, based on the paired-pulse paradigm, facilitates the physiologically correct interpretation of the response threshold values in the different body positions. Values as found in the supine and standing position were the respective threshold intensities that recruited an appropriate number of afferent fibers to evoke a measurable PRM reflex. Those identified in the prone position were the (underestimated) thresholds for evoking a reflex or direct motor response. Because the response threshold was higher in prone than in the other body positions, the afferent stimulation threshold had to be higher as well, irrespective of whether afferent or efferent fibers were recruited at a lower stimulation intensity in prone position.

### Biophysical influences

The body position influences the geometry of the volume conductor in-between the stimulating electrodes and the relative position of the target neural structures within the generated electric field. Specifically, the bony structures of the spine influence the local current densities [40]. Most of the current flows outside the spine, while some current passes the spinal canal mainly via the electrically better conducting soft tissues [41]. The percentage of the total current flow through the spinal canal [39] may vary because of changes of the spinal curvature and geometrical changes of the soft tissues.

The transverse location of the spinal cord in the spinal canal depends on the body position [42,43]. When subjects are turned from the supine to the prone position, the spinal cord migrates by approximately 2.2 mm in anterior direction at the T11 vertebral level, and by 3.4 mm at T12 [42]. These changes considerably influence the effect of spinal cord stimulation when utilizing epidural electrodes, because they increase the distance between a postero-medially placed bipolar epidural electrode to the spinal cord by 61% and 113% at the T11 and T12 vertebral levels, respectively. On the other hand, the relative increase of the distance between the paravertebral stimulating surface electrode and the spinal cord in the present study is small and the absolute values of the electric potential around the spinal cord will be negligibly affected, since the increased distance is attributable to the relatively well conducting cerebrospinal fluid in which the voltage drop (i.e., potential gradient or the electric field) is smallest when the electrical field directionality is transversal. Thus, we conclude that the mere change of electrode-to-spinal cord distance between supine and prone positions does not cause the observed response nature change.

The activating-function concept states that the second-order spatial derivative of the electric potential along the fiber’s trajectory represents virtually injected currents by extracellular stimulation [44–46]. Thus, the nerve fiber path in the field (e.g., bends) and transitions of the fiber between anatomical tissues with different electrical conductivities create sites of de- and hyperpolarization. These then determine the activation thresholds. Computer simulations showed [39,41,47,48] that in transcutaneous spinal cord stimulation there are three such stimulation hot-spots: at the entrance of the posterior root sensory fibers into, at the exit of anterior root motor fibers from the spinal cord, and at their common transition from the spinal canal into the intervertebral foramina. For selective sensory nerve fiber stimulation the first hot-spot must have the lowest threshold. We propose that the migration of the spinal cord in the canal from supine to prone [42] influences the root-fiber trajectories (cf. [43]) in a way that the threshold of this hot-spot becomes equal or larger than those of the others. Thus, the changes of root-fiber trajectories between prone and the other positions impede sensory and favor motor fiber stimulation. Imaging studies combined with computer simulations are needed to verify this hypothesis and could help to develop derivations of the present method to transcutaneously stimulate sensory roots independently from the body position.

The lower threshold intensities in standing compared to the supine position might be caused by movement of the skin on the back that in turn would alter the rostro-caudal position of the paravertebral electrodes. The changed body position might also influence the rostro-caudal position of the lumbosacral spinal cord in the spinal canal. Both factors potentially influence threshold intensities [11–13]. We suppose that biophysical rather than central effects caused the threshold differences, because the soleus H reflex threshold normalized to that of the M-wave has been previously shown to be constant between supine, seated and standing positions for both spinal cord intact and injured individuals [49].

### Central influences during standing

The nature of the responses in prone position (motor fiber activation bypassing the spinal cord circuitry) and the absence of a volitional or postural task suggest that the differences between prone and supine positions are largely caused by biophysical changes. Yet, in standing, vestibular and peripheral sensory inputs from cutaneous and muscular receptors modulate the reflexes [50–53]. The soleus H reflex amplitudes are suppressed during standing when compared to sitting, prone and standing with back support [52,54–56]. This suppression when standing without in comparison to with support is caused by presynaptic inhibition of the Ia afferents onto the motoneuron pools [54]. Here, similarly, triceps surae PRM reflex amplitudes were reduced by ~50% between supine and standing. Those of the other muscles were also reduced but by a lesser degree.

## Conclusions

Transcutaneous spinal cord stimulation selectively activates sensory fibers in supine and standing individuals while in the prone position, motor fibers are predominantly activated. Thus, it is a viable tool for evoking test reflexes for neurophysiological studies of the human lumbar spinal circuitry as well as for providing tonic sensory drive for neuromodulation applications when used in supine or upright positions.
